# Translation and validation of the Nature Relatedness Scale to German

**DOI:** 10.3389/fpsyg.2024.1507983

**Published:** 2024-12-16

**Authors:** Viviane Gallus, Christine Ida Hucke, Katja Butter, Martin Ohlmeyer, Christoph van Thriel

**Affiliations:** ^1^Department Neurotoxicology and Chemosensation, Leibniz Research Centre for Working Environment and Human Factors, Dortmund, Germany; ^2^Human Health and Consumer Protection, Johann Heinrich von Thünen Institute of Wood Research, Hamburg, Germany

**Keywords:** questionnaire translation, confirmatory factor analysis, exploratory factor analysis, nature relatedness, nature connectedness, environmental attitudes

## Abstract

Over the recent past, tools have been developed to asses people's connection to and attitudes towards nature due to increasing interest in this topic in society and research. We translated one such questionnaire, the Nature Relatedness Scale, consisting of three subscales (NR-Self, NR-Perspective, NR-Experience) to German. We collected 251 data sets and performed a confirmatory factor analysis, followed by an exploratory factor analysis. The analyses revealed that the reliability of the German NRS as a whole was good. However, they also showed NR-Perspective to be the weakest factor, and that NR-Self was a rather vague construct, closely connected to the other two subscales. Overall, we came to the conclusion that the NRS' three subscales are not as distinct and reliable as expected, and instead suggest a two-factor solution (NR-Presence and NR-Perspective) for use in German.

## 1 Introduction

The Nature Relatedness Scale (NRS) was developed to ask about one's connection to nature and the environment (Nisbet et al., 2009). The original scale consisted of 30 candidate items on which 831 Canadian participants rated themselves. Additionally, in a second phase, a subsample of 184 participants answered questions on vegetarianism, pet ownership, consumer habits, environmental activism, and outdoor activity habits and filled in various other pre-existing scales measuring e.g., environmentalism, biophilia, and the big five personality traits. Through an exploratory factor analysis on the data yielded by the original, larger sample, the authors came to the conclusion that three distinct factors underlie an overall scale of 21 items: Nature Self (NR-Self), Nature Perspective (NR-Perspective), and Nature Experience (NR-Experience). The authors described NR-Self as one's identification and “personal connection to nature” (Nisbet et al., 2009) and included nine items. NR-Perspective was summarized as an attitude toward the environment and humans' effects on it and contained six items. Finally, they summarized NR-Experience as one's comfort in the outside and being “drawn to the wilderness” (Nisbet et al., 2009). These three factors accounted for 34.18% of the total variance. The remaining nine items were dropped. Since the authors expected these three subscales to be interrelated and thus correlated, they fitted an oblique Promax rotation (κ = 4). The overall internal consistency was high, with a Cronbach's α of 0.87 (NR-Self: 0.87, NR-Perspective: 0.66, NR-Experience: 0.80). Additionally, the test-retest reliability over 6–8 weeks was high (NR: 0.85, NR-Self: 0.81, NR-Perspective 0.65, NR-Experience: 0.85). Differences in measured NR could also predict the behavioral differences regarding vegetarianism, pet ownership, consumer habits, environmental activism, and outdoor activity habits in the subsample, lending credibility to construct validity. Similarly, there were positive correlations between NR and the environmental attitudes measured through other scales. Now, with the NRS thus established in English, the intent of our study was to create a German translation of the questionnaire to use for studies conducted in German. This is necessary because the topic has been of increasing interest in research in recent years, as can be seen by comparing topic-related article keywords between 2008 (the year the original study was first published online) and 2021 (the year of our data collection) (Table 1). Additionally, a replication in German can also produce further information about the original NRS, e.g., if the understanding of Nature Relatedness is culture-specific or otherwise tied to language.

**Table 1:** Comparison of article keywords on Pubmed by year.

**Keyword**	**2008**	**2021**
Environmental awareness	833	3,433
Environmental attitudes	1,945	2,835
Nature relatedness	64	247
Nature connectedness	9	107
Environmentalism	2	47

To summarize, our goal was to translate the scale, including all three subscales, into German, and subsequently replicate the original factor structure with an independent sample to confirm its construct validity. We expected that our translated items match the same factor structure as the original data.

## 2 Method

### 2.1 Sample

The only criteria for participation were being over 18 and good German language proficiency, although it was not necessary for them to be native speakers. Participants were recruited online through university but did not have to be university students themselves. Data were collected via Tivian's survey system Unipark. Two hundred and fifty-one complete sets of responses were collected (no missing values), with 49 men, 198 women, and 4 participants identifying differently. Their mean age was 25.725 (*SD* = 14.255, minimum 18).

### 2.2 Questionnaire measurement

We chose a native German speaker with high English proficiency to conduct an initial translation of the NRS items. They were assisted by a native English speaker who weighed in on the precise meaning of items as they were not always unambiguous. The most contentious was item 12, “Animals, birds and plants have fewer rights than humans”, which can have an either prescriptive or descriptive reading. While it was clear from this item being inverted that it was meant as a prescriptive statement, we opted to keep the ambiguity in our translation to stay true to the original phrasing and produce similar impressions in participants. Similarly, some items included wording that has no direct equivalent in German, which was then substituted by multiple words, e.g., “wildlife” was thus paraphrased by “Wildtiere und -pflanzen” (wild animals and plants). The initial translation was then reviewed and adjusted by two other native German speakers for clarity (Table 2). Participants filled in the questionnaire on a five point Likert scale online, one meaning complete agreement, and five meaning complete disagreement.

**Table 2:** Translated items and expected factor structure.

**Factor**	**Item**	**Label**	**English**	**German**	**Mean (SD)**
NR-self	1	v19	My connection to nature and the environment is a part of my spirituality	Meine Verbindung zur Natur und Umwelt ist Teil meiner Spiritualität	3.008 (1.23)
	2	v24	My relationship to nature is an important part of who I am	Meine Beziehung zur Natur ist ein wichtiger Teil meiner Identität	2.598 (1.114)
	3	v25	I feel very connected to all living things and the earth	Ich fühle mich sehr verbunden zu allen Lebewesen und der Erde	2.526 (0.973)
	4	v26	I am not separate from nature, but a part of nature	Ich bin nicht getrennt von der Natur, sondern ein Teil von ihr	2.159 (0.933)
	5	v27	I always think about how my actions affect the environment	Ich denke immer darüber nach wie meine Handlungen die Umwelt beeinflussen	2.371 (0.868)
	6	v28	I am very aware of environmental issues	Ich bin mir Umweltthemen sehr bewusst	2.147 (0.843)
	7	v29	I think a lot about the suffering of animals	Ich denke viel über das Leid von Tieren nach	2.41 (1.001)
	8	v30	Even in the middle of the city, I notice nature around me	Selbst mitten in der Stadt bemerke ich die Natur um mich herum	2.267 (0.923)
	9 (-)	v31	My feelings about nature do not affect how I live my life	Meine Gefühle der Natur gegenüber beeinflussen nicht wie ich mein Leben lebe	2.426 (0.924)
NR-perspective	10 (-)	v32	Humans have the right to use natural resources any way we want	Menschen haben das Recht natürliche Ressourcen zu benutzen, wie wir wollen	1.904 (0.857)
	11 (-)	v37	Conservation is unnecessary because nature is strong enough to recover from any human impact	Naturschutz ist unnötig, weil die Natur stark genug ist sich von jedwedem menschlichen Einfluss zu erholen	1.371 (0.7)
	12 (-)	v38	Animals, birds and plants have fewer rights than humans	Tiere, Vögel und Pflanzen haben weniger Rechte als Menschen	2.375 (1.231)
	13 (-)	v39	Some species are just meant to die out or become extinct	Manche Spezies sind einfach dazu bestimmt auszusterben	1.92 (1.009)
	14 (-)	v40	Nothing I do will change problems in other places on the planet	Nichts was ich tue wird die Probleme in anderen Teilen der Welt verändern	2.191 (0.973)
	15	v41	The state of nonhuman species is an indicator of the future for humans	Der Zustand von nicht-menschlichen Spezies ist ein Indikator für die Zukunft der Menschheit	2.359 (0.979)
NR-experience	16 (-)	v42	The thought of being deep in the woods, away from civilization, is frightening	Der Gedanke tief im Wald und weg von der Zivilisation zu sein ist beängstigend	2.458 (1.096)
		17	v47	My ideal vacation spot would be a remote, wilderness area	Mein idealer Urlaubsort wäre ein ferner Ort in der Wildnis	2.721 (1.089)
		18	v48	I enjoy being outdoors, even in unpleasant weather	Ich genieße es draußen zu sein, selbst bei schlechtem Wetter	2.247 (0.989)
		19 (-)	v49	I don't often go out in nature	Ich gehe nicht oft nach draußen in die Natur	2.116 (1.065)
		20	v50	I enjoy digging in the earth and getting dirt on my hands	Ich genieße es in der Erde zu graben und Dreck an die Hände zu bekommen	3.187 (1.121)
		21	v51	I take notice of wildlife wherever I am	Ich bemerke Wildtiere und -pflanzen wo immer ich bin	2.502 (1.025)

### 2.3 Data analysis

We conducted most of our analyses in RStudio (RStudio Team, 2021), reading in data by using readxl (Wickham and Bryan, 2019). We began by reverse-scoring appropriate items: items 9–14, 16, and 19.

We tested for multivariate normality using the “MVN” package (Korkmaz et al., 2014) and came to the conclusion that our data did not follow a normal distribution with the Henze-Zirkler test being significant (*HZ* = 1.007, *p* < 0.05). Then, we conducted an unconstrained confirmatory factor analysis (CFA) in RStudio, using the “lavaan” package (Rosseel, 2012), figures having been created with “lavaanplot” (Lishinski, 2024). Items were sorted into the same factors as in the original study: NR-Self consisted of items 1–9, NR-Perspective consisted of items 10–15, and NR-Experience contained items 16–21 (Nisbet et al., 2009). Since the data was not normally distributed, we used the maximum likelihood with robust standard errors (MLR) estimator, which is robust to the breach of this assumption and also yields more reliable results at a small sample size (Yilmaz, 2019). Our confirmatory factor analysis used the χ^2^-distribution and an α-level of 0.05, testing if the factor structure differed significantly from the implied model. We also included several criteria that evaluate the fit of the model: the Comparative Fit Index (CFI), Tucker-Lewis Index (TLI), Root Mean Square Error of Approximation (RMSEA), and Standardized Root Mean Square Residual (SRMR). The two incremental fit indices indicate whether a model is a better fit to the data than the baseline model of all variables being independent (Shi et al., 2019). The RMSEA estimates how close our model's covariance matrix is to the true model's covariance matrix (Shi et al., 2019), and the SRMR is a standardized measure for average residual covariance, comparing the hypothesized model to our sample (Shi et al., 2018). We chose 0.95 as the cut-off value for the CFI, 0.95 for the TLI, 0.06 for the RMSEA, and 0.08 for the SRMR (Hu and Bentler, 1999). The analysis was run a second time, excluding participants who interrupted questionnaire completion or took longer than three standard deviations beyond the mean time. To gain more information about internal consistency, we also calculated a Cronbach's α across all subscales and for each subscale using the “ltm” package (Rizopoulos, 2006) and McDonald's ω using “semTools” (Jorgensen et al., 2022). Items with a low reliability (*R*^2^ < 0.2) were successively removed starting with the lowest *R*^2^. After each item removal, the analysis was rerun.

To gain further information about the factor structure and their items, we followed the CFA with an exploratory factor analysis (EFA). A Bartlett's test for sphericity was employed to ensure that data were not uncorrelated using the “psych” package (William Revelle, 2023). For the subsequent EFA, we used the same Promax-rotation of κ = 4 as the original study did (Nisbet et al., 2009). The number of factors extracted was based on a scree plot and a parallel analysis scree plot (factor extraction method being principal axis factor analysis), which plots factors' eigenvalues against simulated data (William Revelle, 2023). The parallel analysis scree plot was created using “ggplot2” (Wickham, 2016), based on a script provided publicly (Sakaluk and Short, 2017). Item factor loadings were calculated using the “psych” and “GPArotation” package (William Revelle, 2023; Bernaards and Jennrich, 2005), and items were dropped successively until none were *R*^2^ < 0.4.

## 3 Results

### 3.1 Descriptive statistics and data quality

Participants of the 251 complete sets used the full range of the Likert scale (1 − 5) while filling out the NRS, with no missing values. The overall mean score achieved was 2.346 (*SD* = 1.07), and individual mean scores ranged from 1.1 to 3.86. NR-Self had a mean score of 2.435 (*SD* = 1.015), NR-Perspective had a mean score of 2.02 (*SD* = 1.03), and experience 2.539 (*SD* = 1.119). Three participants took a break during testing, the remaining participants took on average 5 minutes (*SD* = 3.598).

### 3.2 CFA

The results of the initial CFA were significant [$\chi_{\left(186\right)}^{2}=393.815,\;p<0.05$], meaning the null hypothesis that the structures specified by the model and produced by the data are the same could not be confirmed. Both incremental fit indices (*CFI* = 0.829 and *TLI* = 0.807) and the *RMSEA* of 0.067 were not sufficient to support the idea of the model either. However, the *SRMR* of 0.074 indicated an acceptable fit. Rerunning the analysis without completion time outliers yielded marginally different results, but not nearly different enough to warrant a change in decision regarding the model. For further analysis, we thus included all participants. The Cronbach's α of 0.84 showed good internal consistency across all items, similarly to McDonald's ω of 0.843.

**Figure 1:**
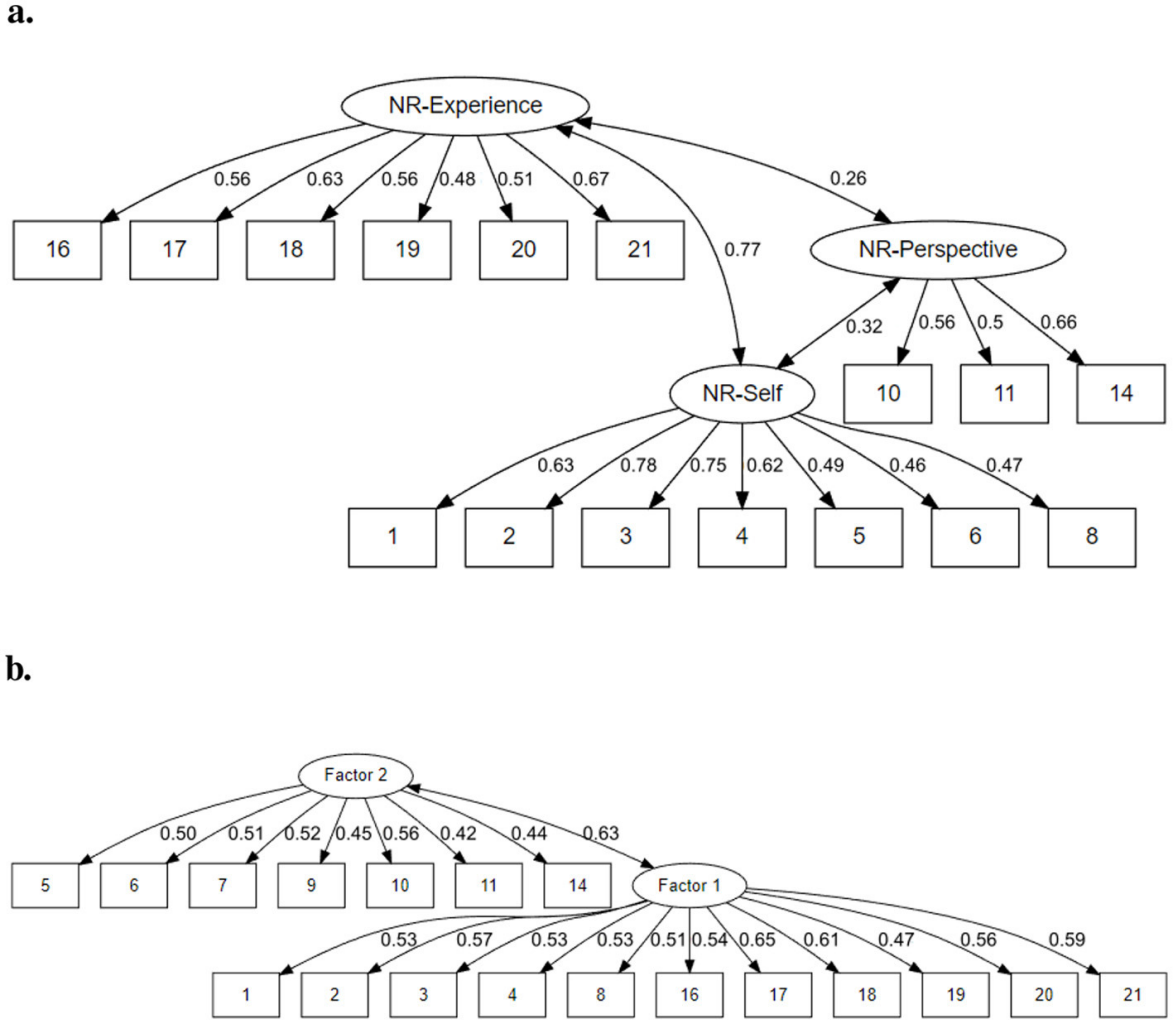
Path diagrams depicting both the **(A)** factor loadings and covariances of the CFA after item removal and **(B)** factor loadings and covariance of our EFA solution, with two factors and after item removal. Factor 1 was later labeled NR-Presence, and factor 2 was labeled NR-perspective.

Items 15, 13, 12, 7, and 9 were then removed in this order, only leaving items with an *R*^2^>0.2. This removal kept CFA-results significant [$\chi_{\left(101\right)}^{2}=233.973,\;p<0.05$], meaning that the structures specified by the model and produced by the data were still not the same. Both incremental fit indices (*CFI* = 0.867 and *TLI* = 0.842) improved but were still not sufficient to support the idea of the model. The *SRMR* of 0.068 remained in an acceptable range and improved slightly. The *RMSEA* of 0.072 stayed outside of an acceptable range. The loadings of the observed variables onto the latent factors can be seen in Figure 1A. The average variance extracted was 0.396 for NR-Self, 0.35 for NR-Perspective, and 0.324 for NR-Experience. The Cronbach's α of 0.836 stayed at good internal consistency across all items, and McDonald's ω was 0.858.

### 3.3 EFA

**Figure 2:**
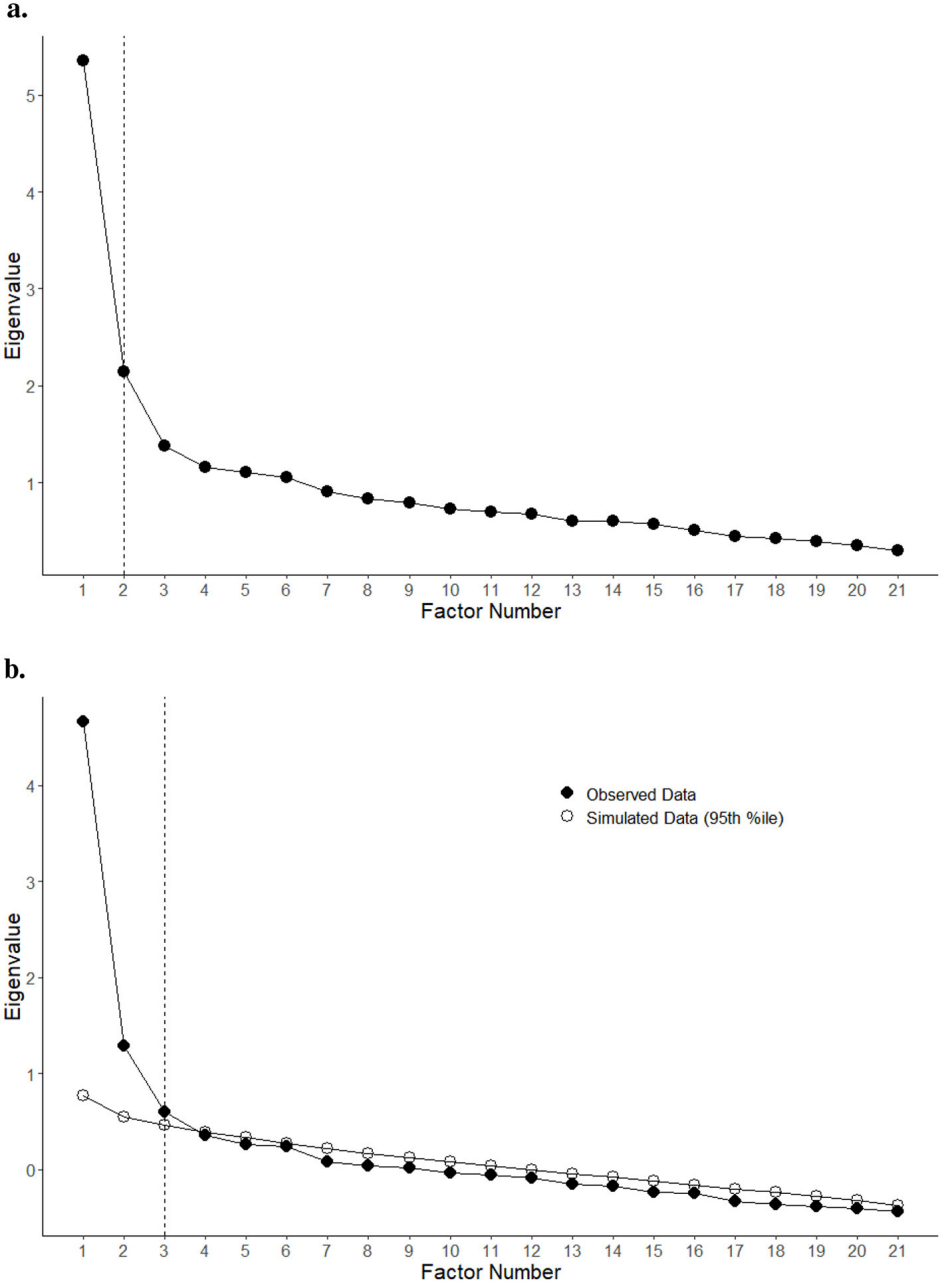
Different approaches to scree plotting produced slightly different results. **(A)** Scree plot of item ratings, suggesting two factors. **(B)** Parallel analysis scree plot of item ratings, suggesting two or three factors. Figures have been generated, using code adapted from Sakaluk and Short (2017).

The first four extracted eigenvalues were 5.356, 2.143, 1.38, and 1.157. Based on scree plots, either two or three factors should be extracted (see Figures 2A, B), but it should be mentioned that the third factor in our sample is much weaker, with a steep drop in explanatory power after the second. We thus arrived at a two-factor solution with some dropped items (12, 13, and 15). Remaining items' factor loadings can be found in Table 3 and Figure 1B. The chosen factor structure explained 32% of the total variance. The intercorrelation between the two factors was 0.44. The overall Cronbach's α was 0.844, and the overall McDonald's ω was 0.879. The first factor had a Cronbach's α of 0.841, and the second factor's α was 0.698, meanwhile the first factor's McDonald's ω was 0.824, and the second factor's was 0.674.

**Table 3:** Factor loadings of EFA for a two factor solution.

**Item**	**Factor 1**	**Factor 2**
1	**0.53**	0.07
2	**0.57**	0.27
3	**0.53**	0.30
4	**0.53**	0.13
8	**0.51**	0.02
16	**0.54**	-0.07
17	**0.65**	-0.11
18	**0.61**	-0.16
19	**0.47**	-0.01
20	**0.56**	-0.12
21	**0.59**	0.15
5	0.16	**0.50**
6	0.15	**0.51**
7	0.02	**0.52**
9	0.14	**0.45**
10	-0.11	**0.56**
11	-0.20	**0.42**
14	-0.01	**0.44**

## 4 Discussion

Using our data, we could not reliably replicate the factor structure of the English NRS with our German translation by means of CFA. While many criteria were on the border of being acceptable, they were not sufficient. Even after removing the items with the weakest *R*^2^, the fit indices were not ideal, and the χ^2^-test stayed significant. However, the SRMR was acceptable, indicating a lower difference between the residuals of our data's covariance matrix and the hypothesized model, and with dropped items both fit indices improved, moving closer to an acceptable range.

In general, the Cronbach's α and McDonald's ω showed that the German NRS as a whole had good internal consistency, similar to that of the original study (Nisbet et al., 2009). This led us to believe that even if the subscales may not apply to our translation, the overall scale is consistent enough to be considered one construct. This of course, left us wondering why we could not replicate the original factor structure. There is the obvious and straight-forward answer that our translation did not convey the same concepts as the original questions. This can be either due to our specific translation or due to general differences in understanding of the questions. Other cultural and ecological differences might also play a role, e.g., the question “The thought of being deep in the woods, away from civilization, is frightening” might be interpreted quite differently by a Canadian vs. a German sample since the two countries differ regarding forests, their inhabiting animals, and inherent dangers. We also believe that some questions like “Animals, birds and plants have fewer rights than humans” can produce very different answers depending on whether the question is interpreted as a descriptive or prescriptive statement. However, this would be less of a language problem, as the same ambiguity might be produced in both languages and, if different between Canadian and German samples, more of an issue of general cultural understanding of the sentence. Another factor may be differences between the samples. Our participants were a little older and had a large range of ages, and while both studies had more women participating than men, ours had relatively even more. Additionally, education levels may have differed. The Canadian sample consisted of undergraduate students, while we did not assess our participants' education. Since they were recruited through university, it is likely that many were enrolled students, but not necessarily all of them. As of now, we cannot say exactly what influence such differences in sample may have had on their respective responses, however, it is entirely possible that these factors contributed to a different understanding of items, or a different relation to the topic at hand, in general.

Most notably, NR-Perspective had the most items removed due to weak factor loadings, according to CFA. Differences between samples may account for some of this factor's weakness. The original sample was surveyed in 2008 while ours was surveyed in 2021. Over this time, attitudes toward nature and environmentalism, as captured by NR-Perspective, may have changed because pro-environmentalist sentiments keep becoming increasingly present in media, fictional or otherwise (Seelig, 2019). Considering mean item values, the three items with the lowest ratings were originally ascribed to this scale (items 11, 10, and 13, Table 2), and NR-Perspective had the lowest overall score as well, meaning highest nature relatedness. Thus, the scale as is may no longer be appropriate to measure interindividual differences in attitude. However, it needs to be mentioned, that the original sample also only produced a Cronbach's α of 0.66 and a retest reliability of 0.65 for this subscale. Our Cronbach's α was of a similar magnitude, casting doubt over the original subscale as well. One of the items that were dropped in our CFA (“The state of nonhuman species is an indicator of the future for humans”), only had a factor loading of 0.17 in the original study as well, so its inclusion is questionable. Taken together, this points toward the possibility that NR-Perspective with its current items never reliably captured the described attitudes to begin with.

To shed further light onto these issues, we conducted an EFA, which had different results than the CFA. Data suggested a two factor solution, rather than three, and three items were dropped, incidentally all items that originally were sorted into NR-Perspective. Additionally, items stemming from NR-Self were now split among the two factors, while NR-Experience and NR-Perspective items did not move. It is noteworthy that in the original study—while NR-Experience and NR-Perspective had no items that loaded meaningfully onto both—many NR-Self items also loaded onto another factor (Nisbet et al., 2009). This leads us to believe that NR-Self may cover a rather vague concept that either underlies and gives rise to the two other subscales or is in turn the distillation and abstraction of the other two, more concrete subscales. Either way, it is not represented in the factor structure found for the German translation. In our structure, factor 1 consisted of NR-Experience items and NR-Self items relating to one's connectedness to nature. Due to the combination not being merely related to physical experiences in nature, we instead suggest the label NR-Presence. Factor 2, which consisted of the three remaining NR-Perspective items and NR-Self items similarly asking for environmental attitudes, may well keep the label NR-Perspective. Cronbach's α and McDonald's ω attest to good overall reliability and decent reliability of the subscales, although NR-Perspective was weaker, similarly to the original NR-Perspective subscale. Additionally, the explained variance was rather low, implying there may have been other, unrecorded influences that affected survey responses.

### 4.1 Limitations

Overall, our approach to replicate the factor structure has its limitations. For example, we did not test participants twice, and thus cannot judge the test-retest reliability of the scale. We also did not use any other external measurement or other scales to compare our data to and further validate the NRS. There are also problems that arise from the translation method itself. We followed an intuitive and semantic approach to capture meanings, but it may have been worthwhile to use a back translation method and piloting to ensure the translated items actually conveyed the same concepts (Papadakis et al., 2022). Additionally, biculturality of the translation group could not be fully achieved since the native English speaker is a US American with German heritage, not a Canadian citizen. This may have negatively impacted the precision of the translation and possibly led to non-equivalence (Papadakis et al., 2022, 2024). Furthermore, our sample does have its differences compared to the original Canadian sample besides their difference in language and cultural background, such as the gender ratio and age range, which may have affected results as well.

### 4.2 Conclusion

In conclusion, we recommend splitting the German translation into two subscales (NR-Presence and NR-Perspective) rather than three, with the caveat that future study should further investigate the reliability and validity of the German NRS by retesting and comparing to other indicators. Additionally, the original, English NRS should also be revisited to further investigate scale-dilution and potential change in NR-Perspective.

## Data Availability

The datasets presented in this study can be found in online repositories. The names of the repository/repositories and accession number(s) can be found at: https://osf.io/aetvg/?view_only=0bffcd9d84554035bd704e957aa1aa4b.
